# Hemodynamic and metabolic recovery in acute myocardial infarction-related cardiogenic shock is more rapid among patients presenting with out-of-hospital cardiac arrest

**DOI:** 10.1371/journal.pone.0244294

**Published:** 2020-12-23

**Authors:** Jakob Josiassen, Ole Kristian Lerche Helgestad, Jacob Eifer Møller, Jesper Kjaergaard, Henrik Frederiksen Hoejgaard, Henrik Schmidt, Lisette Okkels Jensen, Lene Holmvang, Hanne Berg Ravn, Christian Hassager

**Affiliations:** 1 Department of Cardiology, Copenhagen University Hospital, Rigshospitalet, Copenhagen, Denmark; 2 Department of Cardiology, Odense University Hospital, Odense, Denmark; 3 Odense Patient data Explorative Network, University of Southern Denmark, Odense, Denmark; 4 Department of Cardiothoracic Anesthesia, Odense University Hospital, Odense, Denmark; 5 Department of Clinical Medicine, University of Copenhagen, Copenhagen, Denmark; 6 Department of Cardiothoracic Anesthesia, Copenhagen University Hospital, Rigshospitalet, Copenhagen, Denmark; Erasmus Medical Center, NETHERLANDS

## Abstract

**Background:**

Most studies in acute myocardial infarction complicated by cardiogenic shock (AMICS) include patients presenting with and without out-of-hospital cardiac arrest (OHCA). The aim was to compare OHCA and non-OHCA AMICS patients in terms of hemodynamics, management in the intensive care unit (ICU) and outcome.

**Methods:**

From a cohort corresponding to two thirds of the Danish population, all patients with AMICS admitted from 2010–2017 were individually identified through patient records.

**Results:**

A total of 1716 AMICS patients were identified of which 723 (42%) presented with OHCA. A total of 1532 patients survived to ICU admission. At the time of ICU arrival, there were no differences between OHCA and non-OHCA AMICS patients in variables commonly used in the AMICS definition (mean arterial pressure (MAP) (72mmHg vs 70mmHg, p = 0.12), lactate (4.3mmol/L vs 4.0mmol/L, p = 0.09) and cardiac output (CO) (4.6L/min vs 4.4L/min, p = 0.30)) were observed. However, during the initial days of ICU treatment OHCA patients had a higher MAP despite a lower need for vasoactive drugs, higher CO, SVO2 and lactate clearance compared to non-OHCA patients (p<0.05 for all). In multivariable analysis outcome was similar but cause of death differed significantly with hypoxic brain injury being leading cause in OHCA and cardiac failure in non-OHCA AMICS patients.

**Conclusion:**

OHCA and non-OHCA AMICS patients initially have comparable metabolic and hemodynamic profiles, but marked differences develop between the groups during the first days of ICU treatment. Thus, pooling of OHCA and non-OHCA patients as one clinical entity in studies should be done with caution.

## Introduction

Cardiogenic shock is the leading cause of mortality following acute myocardial infarction (MI), and the 30-day mortality rate remains at approximately 50% [1–3]. Most studies assessing mortality among patients with acute myocardial infarction complicated by cardiogenic shock (AMICS) include patients presenting with and without out-of-hospital cardiac arrest (OHCA) [4–6]. OHCA is an independent predictor of mortality after MI [7]. However, the impact of OHCA on short- as well as long-term mortality among patients with AMICS is unclear [8, 9]. The presence of OHCA is increasingly observed among patients with AMICS with recent observational studies reporting a presence > 50% [1, 10], and similar trends are observed in contemporary large randomized controlled trials (RCT) as well [4, 5]. The pathophysiology underlying AMICS with OHCA versus non-OHCA is fundamentally different. AMICS with OHCA is driven by sudden global ischemia followed by post cardiac arrest syndrome (PCAS), while AMICS with non-OHCA is due to progressing myocardial dysfunction leading to low cardiac output (CO) and organ hypoperfusion [11]. Also, studies have shown differences in demographic characteristics of patients with AMICS presenting with and without OHCA [8]. Further, some fundamental aspects of treatment, e. g. targeted temperature management (TTM) differ between the two subgroups. Importantly, severe hypoxic brain injury leading to the withdrawal of life support occurs more frequently among patients with OHCA [12]. To further explore the differences in management and hemodynamic changes during the initial treatment phase in the intensive care unit (ICU), we performed this study in a large contemporary cohort of patients with AMICS presenting with or without OHCA.

## Methods

### Study population

This study is based on the Danish RETROSHOCK cohort [1]. All patients with AMICS were identified through national regulatory registries and underwent individual validation before inclusion in the database. The cohort consists of all patients with AMICS admitted to two tertiary Danish heart centers during the period of 2010–2017, thus representing two-thirds of the Danish population receiving treatment for AMICS.

Based on a screening algorithm, patients diagnosed with either 1) Cardiogenic shock (ICD-10, R57.0), 2) MI as primary or secondary diagnosis (ICD-10, I21.x) (who either died in-hospital and/or was admitted to the ICU and/or treated with vasoactive drugs and/or treated with mechanical circulatory assist device), 3) OHCA as primary or secondary diagnosis (ICD-10, I21.x) (who either died in-hospital and/or was admitted to the ICU and/or treated with vasoactive drugs and/or treated with mechanical assist device), were considered suspicious of AMICS and extracted from the Danish National Patient Registry [1]. A total of 3,553 patients were identified and their medical charts were reviewed by J.J. and O.K.L.H. for individual validation of AMICS and subsequently cohort inclusion.

The MI diagnosis was made at the discretion of the treating physician and based on the fourth Universal Definition of Myocardial Infarction [13].

Presence of AMICS was defined as the fulfillment of all of the following criteria simultaneously; 1) hypotension (systolic blood pressure ≤90mmHg and/or the need of vasopressors and/or mechanical circulatory support), 2) sign(s) of end-organ hypoperfusion (cold/clammy skin, and/or oliguria, and/or altered mental status and/or arterial lactate ≥ 2.5mmol/L) and 3) documented reduction in left and/or right ventricular function due to MI.

Presence of OHCA was defined as cardiac arrest that occurs prior to emergency medical service arrival. Only patients who at some point achieved return of spontaneous circulation (ROSC) were included in the cohort.

### Data collection

Real-time data from the ICU, including basic and advanced hemodynamics, blood gas analyses, medical administrations and ventilator settings, were extracted from the ICU databases used at the at Odense University hospital and Rigshospitalet (Picis clinical solutions and Intellispace Critical Care & Anesthesia respectively). The ICU variables were compared between patients presenting with or without OHCA and are presented as a mean of all patients at defined time-points during the first 72 hours following ICU admission. A calculated mean value is reported in cases where hemodynamic variables were recorded multiple times within an hour. If lactate levels were measured more than once per hour, the highest value measured within that hour was used.

A vasoactive inotropic score (VIS) was calculated as dopamine dose (μg/kg/min) + dobutamine dose (μg/kg/min) + 100 x epinephrine dose (μg/kg/min) + 10 x milrinone dose (μg/kg/min) + 10.000 x vasopressin dose (U/kg/min) + 100 x norepinephrine dose (μg/kg/min) [14].

### Statistics

The cohort of patients with AMICS was stratified into two groups: those presenting with versus without OHCA. Data with Gaussian distribution are presented as mean and standard deviation and compared using an ANOVA test. Data with non-Gaussian distribution are presented as median with 1^st^ and 3^rd^ interquartile range and compared using a Kruskal Wallis test. Dichotomous variables are presented as numbers and percentages and compared using a chi-square test. A multivariable analysis was performed by using a cox proportional hazards model and results are presented as hazard ratio and 95% confidence interval (CI).

A Kaplan-Meier approach was used to estimate the 30-day mortality rates, and a log-rank test was used to compare differences between groups.

All ICU variables were compared using repeated measurement mixed models for unstructured covariance structure and the patients stratified into 2 groups: OHCA and non-OHCA. The two groups were compared, including the interaction over time during the first days of ICU admission. All statistical analyses were done using SAS® Enterprise software (version 7.1 SAS Institute, Cary, North Carolina, USA). All figures are made with GraphPad Prism 7.0.

### Ethics

Approval for current study was granted by the Danish Patient Safety Authority (ID: 3-3013-1133/1) and the Danish Data Protection Agency (ID: 16/7381 and 18/23756).

## Results

From 2010–2017, a total of 1,716 patients with AMICS were identified and included in the cohort. Of these, 723 (42%) presented with OHCA (median time to ROSC, 20 mins (Q1-Q3: 14–30 mins)). OHCA patients were younger (63 years vs 70 years), more frequently male (85% vs 67%), and more often sedated upon ICU admission (95% vs 35%), Tables 1 and 2. OHCA patients initially had higher lactate levels (6.2 mmol/L vs 5.0 mmol/L) and left ventricular ejection fraction (LVEF) (30% vs 25%) compared to non-OHCA patients (p<0.0001 for all). All AMICS patients underwent coronary angiography immediately upon arrival and revascularisation accordingly depending on futility assessment by the heart team in the cath lab. From the complete cohort of 1,716 patients, 89% of OHCA patients and 85% of non-OHCA patients were acutely revascularized (p = 0.02). Among patients surviving to the ICU, no difference were seen among subgroups (p = 0.84).

**Table 1:** Patient characteristics.

Patient characteristics				
	OHCA	non-OHCA	p	Missing, n
Number of patients, n (%)	723 (42)	993 (58)		0/1716
Age, mean (SD)	63 (12)	70 (12)	<0.0001	0/1716
Sex (male), n (%)	612 (85)	662 (67)	<0.0001	0/1716
Hypertension, n (%)	305 (45)	538 (57)	<0.0001	95/1716
Dyslipidemia, n (%)	205 (30)	352 (38)	0.0024	112/1716
Diabetes mellitus, n (%)	99 (15)	220 (23)	<0.0001	90/1716
History of myocardial infarction, n (%)	81 (12)	171 (18)	0.0005	70/1716
History of COPD, n (%)	50 (7)	127 (13)	0.0001	96/1716
History of stroke, n (%)	45 (7)	91 (10)	0.0295	92/1716
Known peripheral artery disease, n (%)	34 (5)	107 (11)	<0.0001	96/1716
Known ischemic heart disease, n (%)	146 (21)	335 (35)	<0.0001	68/1716
Admission findings				
Systolic blood pressure, mean (SD)	84 (16)	81 (15)	0.0014	65/1716
Diastolic blood pressure, mean (SD)	53 (12)	52 (12)	0.0540	152/1716
Heart rate, mean (SD)	81 (22)	90 (27)	<0.0001	232/1716
Adm. lactate concentration (mmol/L), median (Q1;Q3)	6.2 (3.5;10.6)	5.0 (2.9;8.2)	<0.0001	320/1716
Left ventricular ejection fraction, median (Q1;Q3)	30 (20;40)	25 (15;35)	<0.0001	99/1716
Atrial fibrillation requiring intervention	129 (18)	254 (26)	0.0001	12/1716
Ventricular fibrillation during admission, n (%)	193 (27)	287 (29)	0.33	14/1716

Adm.: Admission. COPD: Chronic obstructive pulmonary disease. OHCA: Out-of-hospital cardiac arrest.

**Table 2:** Procedural characteristics.

Procedural characteristics				
	OHCA	No OHCA	p	Missing, n
Mechanical ventilation during ICU admission, n (%)	692 (96)	608 (62)	<0.0001	5/1716
Duration of mechanical ventilation (days), median (Q1;Q3)	3 (1;6)	2 (1;6)	0.0003	5/1716
Renal replacement therapy, n (%)	131 (19)	242 (24)	<0.0001	3/1716
Temporary pacing	89 (12)	210 (21)	<0.0001	17/1716
Mechanical circulatory assist device				
IABP, n (%)	38 (5)	150 (15)	<0.0001	4/1716
Impella, n (%)	76 (11)	133 (13)	0.27	3/1716
VA-ECMO, n (%)	22 (3)	36 (4)	0.59	5/1716

IABP: Intra-aortic ballon pump. ICU: Intensive care unit. VA-ECMO: Veno-arterial extracorporeal membrane oxygenation.

**Fig 1:**
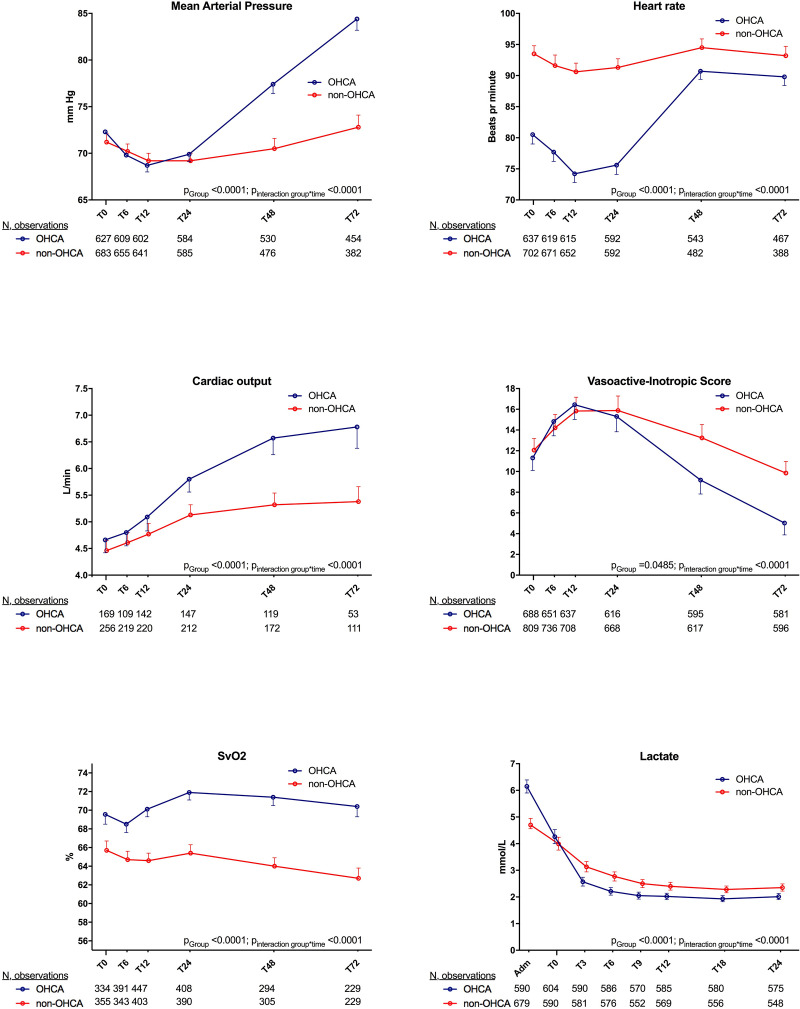
Physiologic parameters and calculated VIS score doses during the first 72 hours after intensive care unit admission among the patients who were admitted to the ICU (n = 1532) are stratified into subgroups presenting with and without out-of-hospital cardiac arrest. OHCA: Out-of-hospital cardiac arrest. Lactate levels are depicted from hospital admission and during the first 24 hours of intensive care unit admission.

A total of 1,532 patients were admitted to the ICU. Of the remaining patients 181 patients died before reaching the ICU and 3 patients were admitted to the cardiac care unit. Patients who did not survive to ICU were older (76 vs 66 years) more often non-OHCA patients (89% vs 11%) and they presented with a lower LVEF (20% vs 30%) as well as a higher lactate levels (6.7 mmol/L vs 5.1 mmol/L) (p<0.0001 for all) compared to the patients admitted to ICU. At the time of ICU arrival, no differences were observed between the groups in the hemodynamic and metabolic variables used to define cardiogenic shock, including mean arterial pressure (MAP) (72 mmHg vs 70 mmHg, p = 0.12), cardiac output (CO) (4.6 L/min vs 4.4 L/min, p = 0.30) and arterial lactate (4.3 mmol/L vs 4.0 mmol/L, p = 0.09), Fig 1.

Patients in the OHCA group cleared lactate faster (0.17 mmol/hour vs 0.10 mmol/hour p<0.0001), to a lower level during the first 24 hours of treatment in the ICU compared to non-OHCA group, Fig 1. Additionally, the OHCA group were more often mechanically ventilated and they received a higher dosage of sedation with propofol, especially during the first 24 hours in the ICU when receiving the TTM treatment, Table 3. Consequently, the OHCA group had a lower heart rate (81 bpm vs 90 bpm) at the initiation of treatment in the ICU, Fig 1. During the first 24 hours a comparable therapeutic MAP was targeted among both subgroups, but when looking at the first 72 hours of ICU admission overall OHCA patients had a higher MAP even though they had a lower VIS score, Table 3, Fig 1, S1 Fig. However, there was no difference in the VIS score during the first 24 hours (p = 0.98, sub analysis Fig 1).

**Table 3:** Sedation.

	T0-T72	T0	T6	T12	T24	T48	T72	p_time_	p_group_
**Propofol**	1116								
**OHCA**								<0.0001	<0.0001
Sedated with propofol, n (%)	688 (95)	677 (94)	675 (94)	650 (90)	501 (69)	273 (38)	138 (19)		
Mean dose (SD), milligram/hour		239 (129)	290 (142)	311 (140)	315 (142)	199 (120)	191 (107)		
**non-OHCA**									
Sedated with propofol, n (%)	438 (44)	313 (32)	326 (33)	322 (32)	267 (27)	196 (20)	143 (14)		
Dose, milligram/hour (SD)		178 (122)	200 (133)	199 (129)	195 (119)	165 (102)	152 (94)		
**Midazolam**	147								
**OHCA**								0.02	0.34
Sedated with midazolam, n (%)	46 (5)	8 (1)	24 (3)	28 (4)	23 (3)	17 (2)	9 (1)		
Mean dose (SD), milligram/hour		5.3 (2.8)	4.5 (2.8)	4.4 (2.8)	5.0 (2.6)	4.2 (2.7)	4.6 (3.0)		
**non-OHCA**									
Sedated with midazolam, n (%)	101 (10)	34 (3)	42 (4)	54 (5)	56 (6)	52 (5)	41 (4)		
Mean dose (SD), milligram/hour		4.2 (2.4)	4.8 (2.7)	4.5 (2.2)	4.9 (2.7)	5.8 (2.8)	5.9 (2.9)		

Usage and dosage of sedatives. OHCA: Out-of-hospital cardiac arrest.

A pulmonary artery catheter (PAC) was inserted in 438 patients. Compared to those not receiving a PAC, patients receiving a PAC were younger (64 years vs 67 years, p = 0.002) and more often men (80% vs 75%, p = 0.04). A PAC was equally used among the OHCA and non-OHCA groups (24% vs 27%, p = 0.13). CO, assessed using the PAC, was similar among the two groups at the initiation of treatment. However, during the first 72 hours the OHCA group increased their CO more, Fig 1. Further, patients in the OHCA group had a higher SvO2 at admission and during the first 72 hours compared to non-OHCA group which had a subnormal SvO2 throughout the first 72 hours, Fig 1.

**Fig 2:**
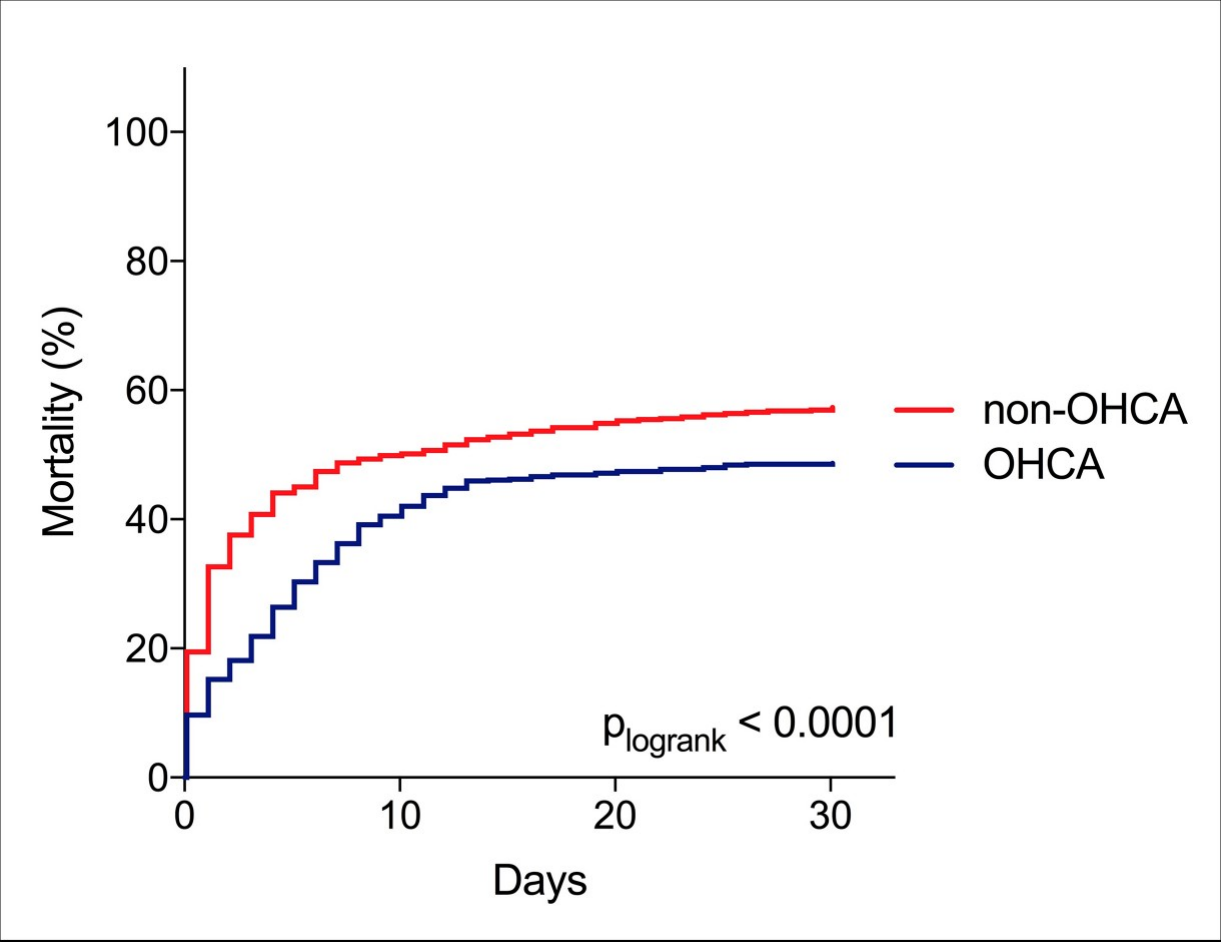
Kaplan Meier curves depicting 30-day mortality among the complete cohort (n = 1716) stratified into subgroups presenting with and without out-of-hospital cardiac arrest. OHCA: Out-of-hospital cardiac arrest.

In terms of overall outcome (n = 1,716), OHCA patients had a lower 30-day mortality compared to patients without OHCA, Fig 2. However, when adjusting for age, this difference disappeared (non-OHCA vs OHCA, HR 1.08 95% CI 0.93–1.265, p = 0.31).

**Fig 3:**
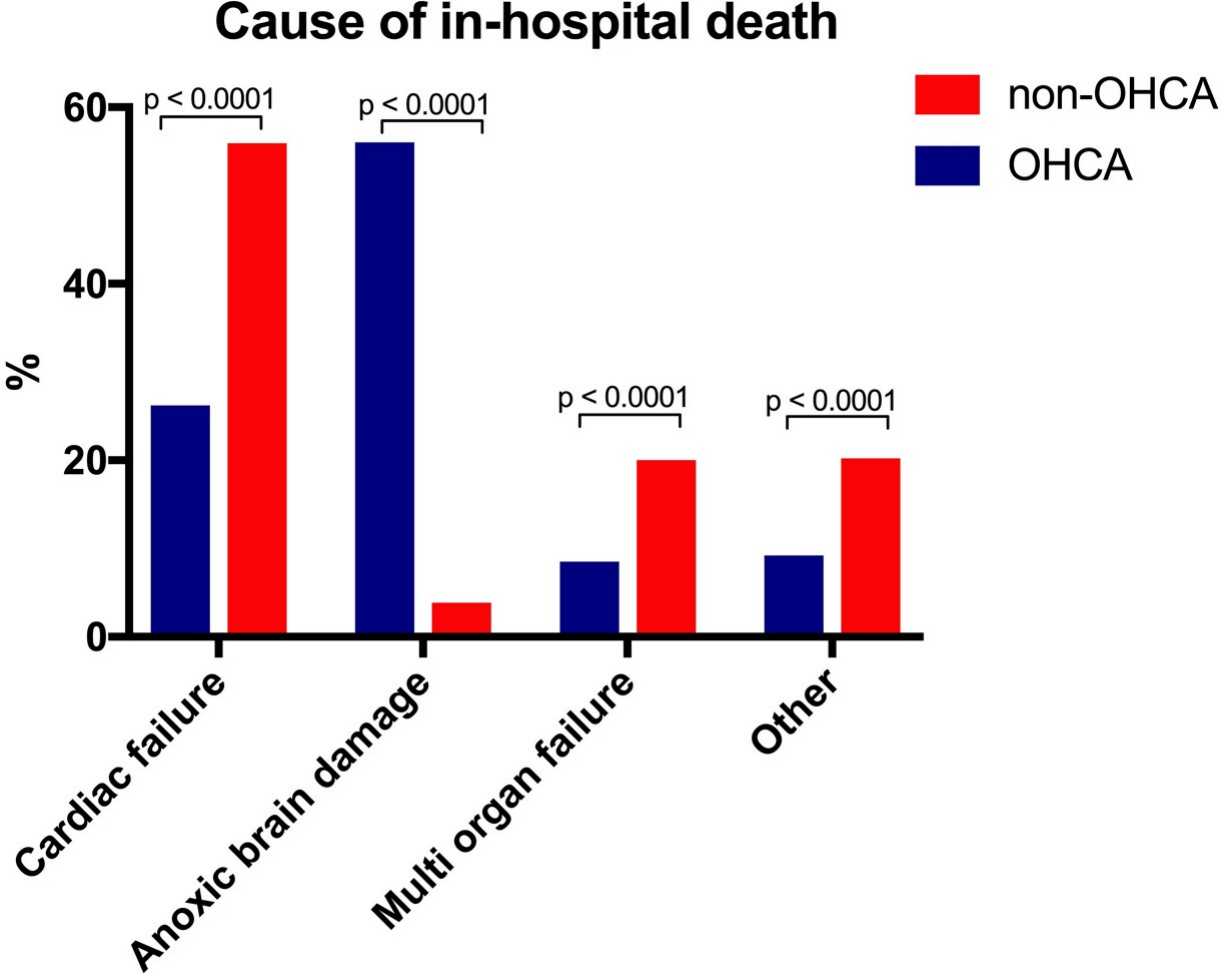
Bar chart depicting cause of in-hospital death among the complete cohort (n = 1716) stratified into subgroups presenting with and without out-of-hospital cardiac arrest. OHCA: Out-of-hospital cardiac arrest.

The cause of in-hospital death differed markedly between the two groups. Hypoxic brain injury leading to withdrawal of life support was the main cause of in-hospital death in the OHCA group (56%) and was only observed in 4% of patients in the non-OHCA group, Fig 3. In contrast, cardiac failure was the main cause of in-hospital death in the non-OHCA group (60%) compared to 27% in the OHCA group, Fig 3.

## Discussion

Despite large differences in patient characteristics and the fact that OHCA patients more frequently were comatose upon admission, OHCA and non-OHCA AMICS patients were clinically inseparable in terms of hemodynamic and metabolic parameters commonly used to define cardiogenic shock at ICU admission. However, during the following days of ICU admission, patients with OHCA improved markedly metabolically and hemodynamically compared to non-OHCA patients and major differences in cause of in-hospital death were seen between the two groups.

### Intensive care unit management

The first 24 hours of treatment in the ICU following immediate revascularization differs among patients with AMICS presenting with versus without OHCA, mainly due to the comatose proportion of OHCA patients receiving TTM treatment [15, 16]. In accordance with the ILCOR guideline recommendations, patients with OHCA received higher doses of sedatives during TTM to maintain the recommended Richmond Agitation-Sedation Scale score of -4 [17]. Since sedatives, especially propofol, have a lowering effect on the blood pressure and heart rate, patients with OHCA treated with sedatives may need vasopressors to maintain an adequate perfusion pressure of 65 mmHg or above. Thus, the need for vasoactive drugs during the initial 24 hours in the ICU in the OHCA group is likely affected by the use of sedatives. Also, the relatively lower HR among patients with OHCA during TTM can be explained by several factors related to the treatment options, including more profound sedation and lower core temperature [18, 19]. It may also be a result of better circulation and hereby less pronounced cardiogenic shock, which is supported by the fact that OHCA patients did not need higher doses of vasoactive and/or inotropic drugs despite markedly higher doses of sedatives. This observation is further supported by a faster lactate clearance.

The lactate level in the early phase of shock is associated with severity of organ hypoperfusion and is a strong predictor of mortality in AMICS [20, 21], and mixed venous oxygen saturation (SvO2) provide information of oxygen extraction and flow (cardiac output) [22, 23]. OHCA was in present study associated with more rapid normalization of lactate and SvO2. Thus, possibly a signal of improved tissue perfusion with decreased lactate production in OHCA after ROSC, whereas lactate levels remained increased in the non-OHCA group due to more prolonged depression of LV function (low SvO2). This may partly explain why patients with OHCA do not have a higher 30-day mortality despite higher lactate levels upon hospital arrival. Better lactate clearance in OHCA patients during the first 24 hours of ICU admission may also reflect improved hemodynamics, which has been associated with improved outcome [24]. A slower lactate clearance following TTM has been observed both in patients with AMICS without OHCA [25, 26]. Since only AMICS patients with OHCA received TTM in the present study, the difference in lactate clearance may be even more pronounced than reported between patients with OHCA versus non-OHCA.

Myocardial stunning is a common part of the post cardiac arrest syndrome during the first hours after ROSC and can easily be interpreted as “classical” AMICS [27–30].

However, a large proportion of these patients quickly recover their cardiac function and hemodynamics after ROSC, which may also be part of the physiological explanation of the faster increase of CO and recovery of SvO2 in the OHCA group.

### Mortality and cause of in-hospital death

The observed unadjusted 30-day mortality of 49% among patients with OHCA and 57% among the non-OHCA patients in this study is comparable to available RCTs and observational studies of patients with AMICS [4, 6, 8]. Available AMICS studies report diverging results on 30-day mortality between OHCA and non-OHCA patients, which may be explained by differences in cardiogenic shock definitions and hereby patient selection [8, 31]. In present AMICS cohort, a younger age was the driver of the lower mortality in the OHCA group. When comparing mortality among OHCA and non-OHCA AMICS patients it is however important to emphasize that OHCA patients reaching the hospital alive are somewhat already highly selected patients, as patients who died prior to hospital admission, cannot be traced in hospital registries. Previous studies on OHCA have shown that, dependent on the underlying rhythm, less than 25% survive to hospital [32, 33]. What can be seen from the present study is that despite impaired hemodynamics and severe metabolic shock upon arrival, patients with OHCA have an equal mortality, but with faster recovery of hemodynamics. In contrast, their outcome is more dependent on neurological recovery, not necessarily relating to hemodynamic stabilization, as marked differences were seen in terms of cause of death. Hypoxic brain injury leading to withdrawal of life support was the leading cause of in-hospital death among OHCA patients, whereas cardiac failure was the main cause of in-hospital death among non-OHCA patients. As far as we are concerned, the cause of in-hospital death among AMICS patients with or without OHCA has not been directly compared previously. However, in the supplementary of the latest published large RCT, the CULPRIT SHOCK trial, cardiac failure and hypoxic brain injury were also the two leading causes of death among patients who died during the first 30 days [4]. Including the results of current study, evidence indicating that OHCA and non-OHCA AMICS patients represent clinical different entities is increasing [34]. It would have large implications on the patient enrollment time, if the subgroups were separated. Consequently, more observational data addressing this are warranted. However, the findings of current study suggest, that future interventions assessed in cardiogenic shock should at least be considered stratified according to whether or not the patient is admitted with OHCA, as one intervention may not have the same effect among clinical entities.

### Limitations

Not all patients survived to ICU admission. The non-survivors were markedly older, and the majority belonged to the non-OHCA group. This may potentially have skewed the observed ICU measurements. However, patients dying prior to ICU admission had higher lactate concentrations and more depressed LVEF, thus representing patients in more profound cardiogenic shock. As the clear majority of these patients were non-OHCA patients, the conclusions of current study are not expected to suffer from over-interpretation.

Hemodynamic instability is one of the main indications for PAC insertion, why this subgroup of patients is suspected of having a worse outcome and somewhat selected.

Finally, this study is observational, with all the limitations applied, including risk of residual confounding and selection bias.

## Conclusion

AMICS patients presenting with and without OHCA are comparable in terms of metabolic and hemodynamic variables used in the classical cardiogenic shock definition at ICU admission. However, during the initial 72 hours extensive metabolic and hemodynamic differences were observed between OHCA and non-OHCA AMICS patients suggesting important underlying differences in the pathophysiology. Future intervention studies in AMICS to optimize circulation should consider stratifying by OHCA, or completely excluding this group of patients, since outcome following OHCA is less likely to rely on hemodynamic stabilization.

## Supporting information

S1 Fig Central temperature and vasoactive drug doses during the first 72 hours after intensive care unit admission. The 1,532 patients who were admitted to the ICU are stratified into subgroups presenting with and without OHCA. During the study period, the protocolized therapeutic hypothermia temperature changed from 33 to 36 degrees Celsius in the OHCA group. Therefore, the average central temperature in the OHCA group is between 33 to 36 degrees during the first 24 hours in the ICU. OHCA: Out-of-hospital cardiac arrest.
(DOCX)
