# Supplementary material for: Hemodynamic and metabolic recovery in acute myocardial infarction-related cardiogenic shock is more rapid among patients presenting with out-of-hospital cardiac arrest
Source: PLoS One. 2020 Dec 23;15(12):e0244294. doi: 10.1371/journal.pone.0244294 (PMC7757873; doi:10.1371/journal.pone.0244294)
Supplement: S1 Fig — The 1,532 patients who were admitted to the ICU are stratified into subgroups presenting with and without OHCA. During the study period, the protocolized therapeutic hypothermia temperature changed from 33 to 36 degrees Celsius in the OHCA group. Therefore, the average central temperature in the OHCA group is between 33 to 36 degrees during the first 24 hours in the ICU. OHCA: Out-of-hospital cardiac arrest. (DOCX) [file pone.0244294.s001.docx]

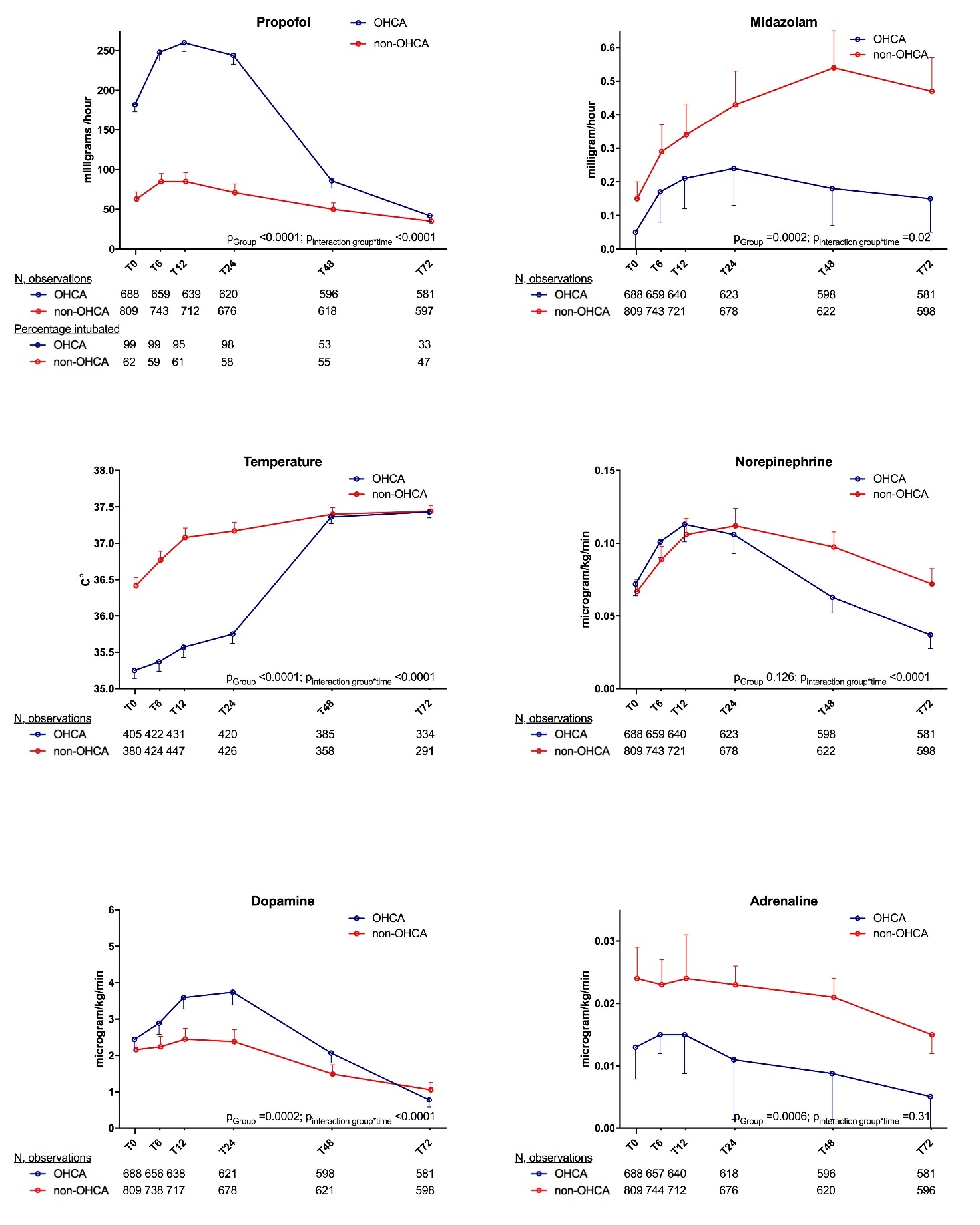


S1 Fig. Central temperature and vasoactive drug doses during the first 72 hours after intensive care unit admission. The 1,532 patients who were admitted to the ICU are stratified into subgroups presenting with and without OHCA. During the study period, the protocolized therapeutic hypothermia temperature changed from 33 to 36 degrees Celsius in the OHCA group. Therefore, the average central temperature in the OHCA group is between 33 to 36 degrees during the first 24 hours in the ICU. OHCA: Out-of-hospital cardiac arrest.
